# Mapping O_2_ concentration in ex-vivo tissue samples on a fast PLIM macro-imager

**DOI:** 10.1038/s41598-020-75928-3

**Published:** 2020-11-04

**Authors:** Rajannya Sen, Alexander V. Zhdanov, Thomaz F. S. Bastiaanssen, Liisa M. Hirvonen, Peter Svihra, Patrick Fitzgerald, John F. Cryan, Stefan Andersson-Engels, Andrei Nomerotski, Dmitri B. Papkovsky

**Affiliations:** 1 School of Biochemistry and Cell Biology, University College Cork, Cork, Ireland; 2 APC Microbiome Ireland, University College Cork, Cork, Ireland; 3 Department of Anatomy and Neuroscience, University College Cork, Cork, Ireland; 4 Centre for Microscopy, Characterisation and Analysis (CMCA), The University of Western Australia, Crawley, WA 6009 Australia; 5 Department of Physics, Faculty of Nuclear Sciences and Physical Engineering, Czech Technical University, 115 19 Prague, Czech Republic; 6 Department of Physics and Astronomy, School of Natural Sciences, The University of Manchester, Manchester, M139PL UK; 7 Irish Photonics Integration Centre, Tyndall National Institute, Cork, Ireland; 8 Physics Department, Brookhaven National Laboratory, Upton, NY 11973 USA

**Keywords:** Biological models, Imaging, Sensors and probes, Medical research, Optics and photonics

## Abstract

O_2_ PLIM microscopy was employed in various studies, however current platforms have limitations in sensitivity, image acquisition speed, accuracy and general usability. We describe a new PLIM imager based on the Timepix3 camera (Tpx3cam) and its application for imaging of O_2_ concentration in various tissue samples stained with a nanoparticle based probe, NanO2-IR. Upon passive staining of mouse brain, lung or intestinal tissue surface with minute quantities of NanO2-IR or by microinjecting the probe into the lumen of small or large intestine fragments, robust phosphorescence intensity and lifetime signals were produced, which allow mapping of O_2_ in the tissue within 20 s. Inhibition of tissue respiration or limitation of O_2_ diffusion to tissue produced the anticipated increases or decreases in O_2_ levels, respectively. The difference in O_2_ concentration between the colonic lumen and air-exposed serosal surface was around 140 µM. Furthermore, subcutaneous injection of 5 µg of the probe in intact organs (a paw or tail of sacrificed mice) enabled efficient O_2_ imaging at tissue depths of up to 0.5 mm. Overall, the PLIM imager holds promise for metabolic imaging studies with various ex vivo models of animal tissue, and also for use in live animals.

## Introduction

Quantitative monitoring of molecular oxygen (O_2_) concentration and its spatial distribution is important for many physiological studies with cells, tissue samples and whole organisms including humans^1–5^. O_2_ is the key substrate and environmental parameter for living systems, and a useful functional readout for respiring samples and their metabolic responses related to oxygen consumption^6,7^. Imaging O_2_ by means of phosphorescent probes and live cell optical microscopy is well suited for this, and has been used increasingly in biomedical research^8,9^. Various phosphorescent probes including cell-permeable or impermeable, small molecule, supramolecular dendrimers, nano- or micro-particulate structures, as well as planar solid-state O_2_ sensors are available^6,9–15^. They allow sensing and imaging of O_2_ in various types of samples in intensity, ratiometric and lifetime-based detection mode, including the time-gated and time-correlated single photon counting (TCSPC) imaging on wide-field, confocal or multi-photon microscopes^6,16^.

Optical O_2_ sensing platform has become an integral part of detailed, high-throughput analysis of cell populations, tissue samples and blood (micro)circulation^1–3,8,17–20^. Phosphorescence Lifetime IMaging (PLIM) is gaining popularity in particular, as it can provide quantitative, accurate, calibration-free 2D/3D/4D mapping of O_2_ concentration in complex biological samples^6,9,16^. Applications of O_2_ PLIM have been demonstrated with in vitro cell and tissue models for metabolic characterisation of neurospheres^21^ and intestinal organoids^22^, analysis of respiration and local O_2_ gradients in ex vivo intestinal and urinary bladder tissue^23,24^, in vivo studies of O_2_ levels and dynamics in animal brain^11,14,25–29^, kidney^30^, bone marrow^31^ and other tissues, including tumour^19^.

At the same time, current PLIM systems have limitations with respect to: (1) the type of samples amenable for analysis; (2) high costs and low flexibility of the imaging equipment; (3) penetration depth, speed and sensitivity of the imaging. Indeed, image acquisition time in PLIM is generally much longer than for FLIM or intensity based imaging^32,33^, particularly in PLIM-TCSPC, which is realised mostly in the slow laser-scanning PMT-based, but not in the camera-based systems. As a result, it takes either many seconds to produce a complete 2D image of relatively small size (~ 30 s for a 256 × 256 pixels using Pt-Glc probe^22^) or sub-seconds to image a bunch of pixel-size reference ROI (0.5 s for 12 micro-ROI using Oxyphor 2P^11^). To achieve maximal acquisition efficiency, water immersion objectives are commonly used. However, when applied for brain imaging through cranial window, this approach may affect the temperature, capillary blood flow of the tissue and its oxygenation state^34^. Imaging of macroscopic samples such as organs and whole animals in PLIM mode is still rare^35–37^; in a standard microscope setup, image area is too small for the analysis of O_2_ dynamics on the organ level.

Many of these limitations have been addressed by the new PLIM-TCSPC macro-imager^38^ based on second generation, intensified Tpx3Cam optical camera^39,40^, which provides single-photon sensitivity, time resolution of 1.6 ns and a readout rate of 80 Mpixel/s^41,42^. The whole imager is compact and flexible like a common photographic camera, and can currently image objects up to 18 × 18 mm with spatial resolution of 39.4 μm^38,43,44^.

In this study, we assessed the analytical performance of this imager with various biological samples, including suspensions of mammalian cells and ex vivo samples of fresh animal tissue and whole organs isolated from sacrificed animals, in conjunction with the well-characterised near-infrared nanoparticle-based O_2_ probe NanO2-IR. The aim was to evaluate the suitability of the imager for in vivo studies with live animals and models of human disease. For that, we performed a series of experiments with phosphorescent staining and imaging of ex vivo tissue samples, and measured intensity and lifetime signals, and O_2_ distribution in various samples and conditions.

## Materials and methods

### Materials

Platinum(II) benzoporphyrin (PtBP)-based O_2_ probe, NanO2-IR, was from Luxcel Biosciences (Cork, Ireland); working solution (1 mg/mL) was prepared by diluting the stock of NanO2-IR (10 mg/mL in H_2_O) with DMEM or McCoy’s 5A media supplemented with 10 mM glucose, 2 mM L-glutamine and 10 mM HEPES (pH = 7.2), immediately prior to use with cells or tissues. DMEM and McCoy’s 5A media, antimycin A (AntA, complex III inhibitor), NaN_3_ (complex IV inhibitor) were from Millipore-Sigma (Burlington, MA). Plasticware was from Sarstedt (Ireland) and Greiner Bio One (Frickenhausen, Germany). CellTiter-Glo ATP Assay kit was from Promega (Madison, WI).

### Tissue culture, analysis of probe toxicity and analysis of cell pellet oxygenation

Human colonic carcinoma (HCT116) and human embryonic kidney (HEK293a) cells were from the European and American Collections of Cell Cultures. HCT116 cells were maintained in McCoy’s 5A medium supplemented with 10% foetal bovine albumin (FBS), 2 mM L-glutamine, 100 U/ml penicillin / 100 µg/ml streptomycin (P/S) and 10 mM HEPES, pH 7.2 (complete McCoy). HEK293a cells were maintained in DMEM supplemented with 10% FBS, P/S and 10 mM HEPES (complete DMEM), both in humidified 5% CO_2_ / 95% air, at 37 °C.

For the probe toxicity testing, cells were seeded on standard 96-well plates pre-coated with collagen IV at 2.5 × 10^4^ cells/well, grown for 24 h, and then exposed to NanO2-IR at 1 mg/ml in complete DMEM for different periods. After that, total cellular ATP levels were measured using CellTiter-Glo Kit, white 96-well plates (Greiner Bio One) and Victor 2 reader (PerkinElmer) under luminometry settings^45^.

In the oxygenation experiments, HCT116 cells grown to 90–100% confluence were trypsinised, washed with DMEM, counted and transferred into 0.5 ml qPCR tubes at 0–5 × 10^6^ cells per tube. Then the cells were precipitated to the bottom by mild centrifugation at 200 g, old media was aspirated, fresh complete DMEM with 1 mg/ml NanO2-IR was added to each tube, cells were re-suspended and precipitated as above. After 30 min incubation at 37 °C tubes with cells were imaged.

### Preparation, staining and treatment of ex vivo animal tissue

All animal procedures were performed under authorisations issued by the Health Products Regulatory Authority (HPRA, Ireland), in accordance with the European Communities Council Directive (2010/63/EU) and approved by the Animal Experimentation Ethics Committee of the University College Cork. Fresh tissue samples were isolated from four-week-old female mice (Envigo, UK). On the day of the experiment, mice were euthanised by decapitation and their whole brain, lungs, fragments of small intestine and colon (large intestine), tail and front paws were quickly dissected, washed with PBS and placed in DMEM media equilibrated at 37 °C.

The selected samples were: tissues with high and low O_2_ consumption (e.g. brain vs lung, or resting vs inhibited tissue); inner and outer areas of hollow organs exposed to air (intestine), and; subcutaneous tissue areas imaged through a thick skin layer (tail and paws). These tissue samples varied in shape and size (~ 8–15 mm, see scale bars in Figures). They were quickly excised, washed with PBS and immediately submerged in complete DMEM pre-warmed to 37 °C. Cortical tissue slices (~ 10 × 10 mm, 1 mm thick) were prepared by dissecting the extracted brain after cleaning; the entire process was completed within 15 min from excision. For the surface staining, the brain, lung and cortical tissue slices were transferred into mini-dishes, submerged in 2 ml of complete DMEM containing 1 mg/ml NanO2-IR and incubated for 30 min at 37 °C. Similar protocol was used for the staining of serosal surface of the intestine (1 mg/ml probe, 15 min). For the intraluminal staining, pieces of intestine were transferred to a dry Petri dish and excess of DMEM was removed with filter paper. Then 1 µl of DMEM containing 1 mg/ml of NanO2-IR was injected into the lumen with a Hamilton syringe, and samples were incubated for 15 min or up to 4 h (time lapse experiments). For the intra-tissue injection, 5 µl aliquots of DMEM containing 1 mg/ml NanO2-IR were injected with a Hamilton syringe subcutaneously into mouse tail or paw, which were pre-incubated for 30 min at 37 °C. After 15 min of further incubation, the samples were imaged under the settings described below.

To inhibit cell respiration in surface-stained tissue samples, they were transferred into Petri dishes with DMEM containing 10 µM AntA and 0.1% NaN_3_, and then incubated for 15 min at 37 °C. Similarly, the intraluminal respiration was inhibited by injecting the AntA/NaN_3_ together with NanO2-IR (1 µg) and incubating the samples for 15 min at 37 °C.

For imaging, all the samples were placed on a fresh and dry Petri dish and any excess liquid was carefully removed with a filter paper. Except for subcutaneous injections, all the experiments were repeated in three times with samples collected on different days. Unless otherwise stated, PLIM experiments were completed within one hour from the start of staining and in less than two hours from excision of tissue samples.

### PLIM Imaging setup

The wide-field PLIM-TCSPC macro-imager based on the time-stamping Tpx3Cam optical camera (Amsterdam Scientific Instruments, The Netherlands) is described in detail elsewhere^38^. Briefly, the compact and flexible configuration of the imager was provided by the Cricket adaptor with integrated S25 image intensifier, power supply and back-end relay optics (Photonis, France), and a 760 ± 50 nm bandpass emission filter (25 mm, Edmund Optics, York, UK). The two C-type lens mounts on the Cricket coupled it with the Tpx3Cam on the one end and the 50 mm Navitar NMV-50M11" lens on the other. A super bright 5 mm red LED (627 nm, Parts Express, Springboro, OH), synchronised with the camera and two pulse generators, was used for pulsed excitation of the sample from the top. For imaging the vials with media and cells, the imager was used in a horizontal orientation, while for tissue samples it was used in an upright orientation. A dry bath/block heater (Thermo Fisher Scientific, Waltham, MA) was used as a heating stage to maintain sample temperature at 37 °C.

All PLIM images were acquired at LED power 4 V, pulse width 50 ns and integration time 20 s. Sophy software (Amsterdam Scientific Instruments) was used to tune the operational parameters, align and focus the system. A custom-designed software was used to acquire the Tpx3Cam raw data in a binary format, while a C-language code was used to post-process the data. The resulting data matrix for each camera pixel was then fitted with a two-exponential function *I (t)* = *A1 exp (-t/τ1)* + *A2 exp (-t/τ2)* in Tri2 software^46^ to determine lifetime values. More details of data processing can be found in^38,47,48^.

### Calibration of NanO2-IR probe

The NanO2-IR probe was calibrated using a handheld lifetime reader OpTech Platinum (Mocon, MN). Standard O_2_–N_2_ gas mixtures were produced by a precision gas mixer (LN Industries, Switzerland). The gas was pumped through a polystyrene tube containing the probe in DMEM and submerged in a circulating water bath (Julabo, Germany) set to 37 °C. The O_2_ concentration was increased stepwise from zero to 21% O_2_ (corresponding to 0–210 µM dissolved O_2_). After ~ 20 min equilibration of the O_2_ concentration and temperature in the sample, lifetime readings were taken at least 5 times to ensure stable and accurate lifetime values.

### Statistics

All independent experiments were conducted in triplicate to ensure consistency of results. Imaging data for cell and tissue samples was analysed using ImageJ software^49^. Statistical analysis of lifetime values obtained from whole PLIM images and derived O_2_ values was performed using R (v3.6.3) and Rstudio (v1.2.5033) software. Lifetime values ranging 19–45 µs were analysed as per the NanO2-IR calibration equation (see Results). Figures were generated using the ggplot2 and patchwork tools. Measurements were aggregated based on experimental treatment and assessed for statistical differences using the Mann–Whitney U test (W- and *p* values were produced). Given the non-normal nature of the data and their unimodal distribution in all groups, differences in the modes (Δ-mode, µs) was deemed the most appropriate metric to report differences between treatments. In Figs. 3e,f, 4g–j and 5, we used three visualization elements that inform on the differences in distribution of the tested parameter between non-normal data sets, namely: (1) the box-plot (shows the median and interquartile range per batch of observations); (2) the violin plot (shows relative density of all observations in a data set with its wideness); the semi-transparent density plot (shows the total relative density of all observations of the same type). BoxPlotR tool (https://shiny.chemgrid.org/boxplotr/) was used to generate ‘boxes-and-whiskers’ plots of the intensity and lifetime variability (in Fig. 3h). Variability of the intensity signals and lifetime values was also evaluated using the standard equation for average deviation: $AveDev = \frac{1}{n}\sum x - x\prime \vee$. For that, all data points along three representative lines across brain samples were normalised to the average values, which were defined as 1 a.u.

## Results and Discussion

### *Set up for the imaging of tissue O*_*2*_

Recently, we have shown that the resolving power of our PLIM macro-imager, which utilizes a 256 × 256 pixels imaging chip, is 12.7 lp/mm^38^. The corresponding line width of 39.4 μm is comparable with the size of animal cells (typically, 10–40 µm in diameter). Thus, high-resolution O_2_ imaging of samples up to 18 × 18 mm in size can be achieved.

To study oxygenation state and O_2_ gradients, which reflect respiration activity of cells, several types of samples including cultured cells, tissue and organs isolated from sacrificed mice were stained with the NanO2-IR probe and then imaged on the imager at 37 °C. For each tissue model, optimised staining (time, temperature, amount of the probe) and imaging (LED power, distance to the sample, signal collection time) protocols were used (data not shown).

Phosphorescent probes used to stain tissue samples respond to local O_2_ concentration by reversibly changing their phosphorescence intensity and lifetime^6,50^. Low lifetime values reflect high O_2_ concentrations (e.g. ~ 210 µM in air-saturated aqueous solution at 37 °C) and low respiratory activity of the sample. Conversely, high lifetime values report on depleted O_2_. Phosphorescence intensity signal depends on probe distribution within cells or tissue sample, staining efficiency, photobleaching, imaging geometry and other factors. Therefore, accurate quantitative imaging of O_2_ concentration in intensity mode is very challenging. In contrast, phosphorescence lifetime-based O_2_ sensing is essentially free from these limitations and hence is regarded as a method of choice for O_2_ imaging^16^.

NanO2-IR probe belongs to the family of cell-permeable nanoparticulate O_2_ probes^51,52^, well characterised and validated with different cell models. The nanoparticle forming RL-100 polymer serves to retain hydrophobic molecules of the phosphorescent dye, provide optimal microenvironment for their quenching by O_2_ molecules and shield from unwanted interferences. The polymer enables solubility of the probe in aqueous solutions, efficient staining of cells and tissues, as well as stable and accurate O_2_ calibration. The high brightness, near-infrared spectral characteristics of PtBP dye (excitable at 620 nm, emits at 760 nm^53^) allows the detection of minute quantities of NanO2-IR and its use in high-resolution O_2_ imaging applications.

### Preliminary experiments

**Figure 1:**
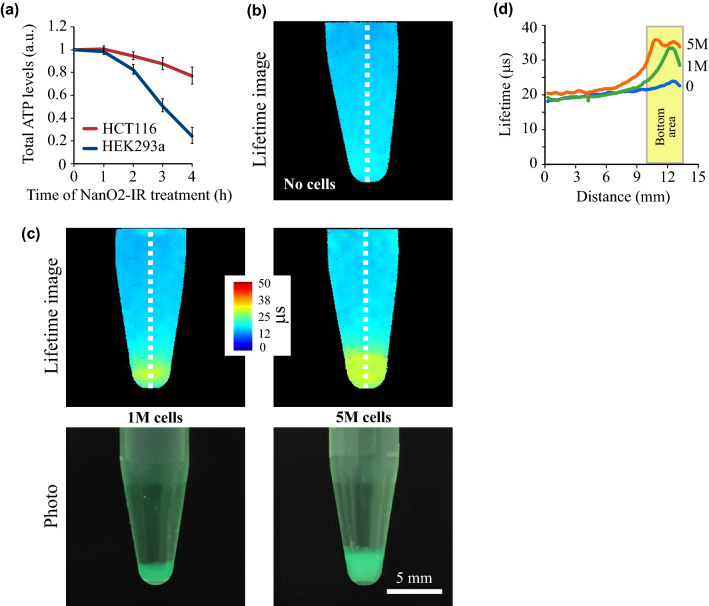
PLIM of dissolved O_2_ distribution in media containing cultured cells. (**a**) Effects of NanO2-IR on viability of HCT116 and HEK293a cells. Total ATP levels were measured with CellTiter-Glo ATP assay after incubating the cells with the probe (1 mg/mL) for indicated time; all values are normalised to ATP levels in untreated cells. N = 3, mean ± SD are shown. (**b**, **c**) PLIM images of the probe (1 mg/mL) evenly distributed in growth medium containing no cells (**b**), and 1 × 10^6^ or 5 × 10^6^ of respiring HCT116 cells precipitated at the bottom; (**c**) photographs of 0.5 mL qPCR tubes with cell pellets. False colour images show increased lifetime values (μs), or decreased O_2_ in and near the cells. (**d**) line profile analysis (along the dotted lines in (**b**) and (**c**)) quantifies this effect.

Staining of cultured cells with nanoprobes based on RL-100 polymer is usually performed at concentrations 5–50 µg/mL and incubation times 3–24 h at 37°C^51,52^. In these conditions probe cytotoxicity is undetectable. However, ex vivo tissue samples are unstable and require faster staining^53^, which can be achieved using higher probe concentrations but may result in significant cytotoxic effects. Using the two common cell lines, HCT116 and HEK293a, we examined cytotoxicity of NanO2-IR at 1 mg/ml concentration in the medium. Analysis of total ATP levels over 4 h exposure period revealed no significant changes in cell viability in the first hour or so, followed by a strong decrease in viability from 2 h onwards (Fig. 1a). Based on this data, we aimed to conduct imaging experiments with live cells and tissues within 1 h from the start of their staining.

Next, we imaged O_2_ in samples containing growth media with different number of live mammalian cells, which were placed in 0.5 mL Eppendorf tubes designed for quantitative PCR. Cells were pelleted at the bottom of the tubes by gentle centrifugation, and gradients of dissolved O_2_ in the surrounding media were generated by subsequent incubation of samples for 30 min at 37 °C. NanO2-IR probe, added at 1 mg/ml final concentration prior to centrifugation, stayed both in the medium and within cell pellet. Figure 1b shows PLIM images of the tubes containing 0, 1 × 10^6^ and 5 × 10^6^ of respiring HCT116 cells. One can see, that sample without cells (left) has uniform colour and almost constant lifetime values, which correspond to air-saturated conditions (~ 210 µM O_2_) across the tube. The minute increase in lifetime at the bottom is most likely due to a temperature gradient within the tube: being equilibrated at 37 °C and placed on the imager stage, its narrow bottom part cooled down faster than the middle and top. In contrast, samples containing 1 × 10^6^ and 5 × 10^6^ cells revealed pronounced O_2_ gradients (Fig. 1c,d) which were cell number-dependent and co-localised with cell pellet. The markedly increased lifetime values inside the pellets at the bottom of the tube reflect depletion of O_2_ due to cell respiration. In the middle and upper parts of the tubes lifetime values are low and do not change much, as they correspond to air-saturated conditions within medium layer, which acts as a barrier for O_2_ diffusion to the cells. Again, the observed lifetime variability in these parts of samples could be attributed to small variations of temperature.

**Figure 2:**
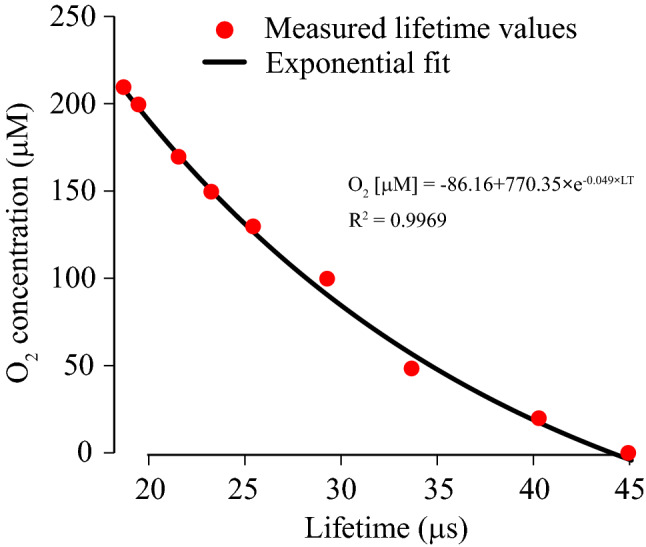
O_2_ calibration of NanO2-IR probe under physiological conditions. Lifetime values were measured with a handheld reader Optech (Mocon, USA) in liquid samples (DMEM media) equilibrated with 0%, 2.0%, 5.0%, 10.0%, 12.0%, 15.0%, 17.0%, 20.0% and 21.0% O_2_ gas standards produced on a gas mixer, at 37 °C. Exponential fitting was performed using https://mycurvefit.com/.

Lastly, O_2_ calibration of NanO2-IR was conducted using a handheld lifetime reader Optech, by measuring phosphorescence lifetime of 1 mg/mL probe dissolved in DMEM medium and equilibrated with known gas mixtures (O_2_ balanced with N_2_), at 37 °C. Figure 2 shows calibration data points, their fitting and the resulting analytical equation: O_2_ [µM] = − 86.16 + 770.35 × e^−0.049×LT^, which can be used to convert measured lifetime values (LT [µs]) into O_2_ concentrations (exemplified in Fig. 4e) and PLIM images into O_2_ maps (Fig. S3).

From these preliminary experiments we concluded that bright phosphorescent probe NanO2-IR and the new Tpx3Cam-based PLIM imager can be applied for accurate measurement of O_2_ in live tissue samples isolated from sacrificed animals, using the once-off calibration function described above.

### Imaging of mouse brain and lung tissue surface-stained with NanO2-IR probe

Due to the ethical and technical constrains associated with the use of live animals, in this study we used samples of tissues freshly isolated from sacrificed animals, in which cells were still alive and respiring, though stressed as their normal supply of O_2_ through vasculature was interrupted. In the ex vivo settings, O_2_ passively diffuses to the tissue samples from air-saturated medium or ambient air, which could impose an additional hypoxia stress to the inner tissue areas. O_2_ solubility, quenching efficiency of NanO2-IR and respiration activity of cells are strongly influenced by temperature^6,50^, therefore temperature of the sample stage was maintained at 37 °C during the experiments. With the above limitations, these tissue models are not ideal for physiological studies, however they were deemed appropriate for the assessment of analytical performance and general suitability of the PLIM imager for in vivo applications.

We imaged different types of animal tissue stained by topical application of NanO2-IR probe. Firstly, we stained three specimens of whole brain cortex and lung with NanO2-IR and analysed them by PLIM. For both tissue types, we used the following staining and imaging protocol. Sample 1 was stained for 30 min and then imaged. Samples 2 and 3 were stained for 15 min, imaged, then treated with respiration inhibitors for 30 min and imaged again under the same settings. After that, sample 1 was imaged again as untreated control, to trace possible changes in tissue oxygenation over the time of the experiment. The settings for acquiring PLIM images of these and other tissue types are given in Table S1.

**Figure 3:**
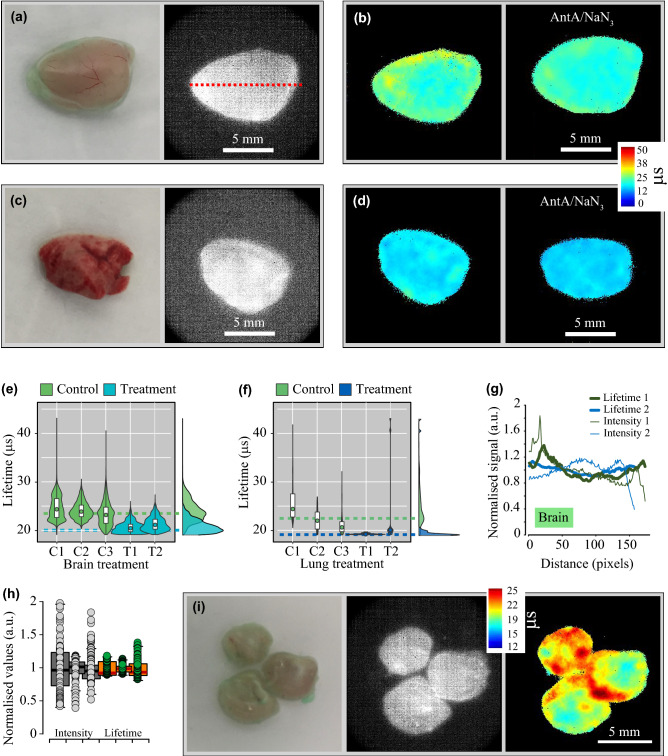
PLIM of O_2_ in the surface-stained mouse brain and lung tissue samples. (**a**) Photograph and phosphorescence intensity image of cortex tissue samples stained with NanO2-IR (1 mg/mL in DMEM, 30 min). (**b**) Corresponding PLIM images of the untreated control (C1-C3) and AntA/NaN_3_-treated samples (T1, T2). (**c**) Photograph and phosphorescence intensity image and (**d**) PLIM images of untreated control (C1–C3) and AntA/NaN_3_-treated lung tissue samples (T1, T2). (**e**, **f**) Lifetime frequency histograms for the brain and lung tissue with normal and inhibited mitochondrial respiration. Violin plots show: on the Y-axis—the distribution of lifetime values; on the X-axis—sample names and relative frequencies, with which lifetime values were recorded. Box-plot partition of the violin plots represent the median (circle) and interquartile ranges. Dashed horizontal lines represent the aggregated modes per tissue type and treatment. On the right side, semi-transparent colour-matched aggregated lifetime distributions are shown. Colours correspond to the predominant spectrum in the PLIM images. (**g**, **h**) Line profile analysis of the variability and interdependence of the intensity and lifetime values across the brain tissue samples (exemplified by a red dotted line in (**a**)); three line profiles in three brain samples were analysed in total. In (**h**), all measured values are shown as single data points normalised to the mean; average deviations (in a.u.) were: for intensity signals—0.287, 0.076 and 0.159; for lifetime values—0.097, 0.057 and 0.124. (**i**) Tissue photograph, phosphorescence intensity of the probe and PLIM images of cortex slices are shown. Scale bars show object dimensions. Rainbow false colours in (**b**, **d**, **i**) are used to visualise the range of lifetime values.

The resulting phosphorescence intensity and PLIM images in Fig. 3a–d reveal efficient surface staining with NanO2-IR for the two tissues. The colours clearly reflect the difference in lifetime values for the two tissue types and, hence, their different oxygenation, which is driven by respiratory activity. In the lung tissue the overall variability of lifetime signals was higher than in the brain due to the elevated values in sample 1, which has no conclusive explanation.

As expected, treatment of the brain tissue with AntA/NaN_3_ caused a reduction in NanO2-IR lifetime (Δ-mode = 3.48 µs), indicating the change in tissue oxygenation from ~ 150 to 200 µM upon the dual inhibition of mitochondrial cytochrome c reductase and oxidase (Fig. 3b,e, Fig. S1a). In turn, the same inhibition of mitochondrial respiration in lung tissue had little effect on lifetime values (Δ-mode = 1.46 µs, Fig. 3d,f). We attribute this to the intrinsically low respiratory activity of the lungs: the initial O_2_ levels in resting tissue were close to air-saturated and could not be further increased by inhibitory treatments. The porous structure of the lung tissue has higher area of gas exchange, thus also contributing to increased tissue oxygenation.

PLIM of different surface-stained samples of the same tissue type (e.g. samples 1, 2, 3 of the brain, Fig. 3e) or the same sample at different time points (sample 1 of the brain, not shown) produced consistent lifetime values with moderate variability, which was lower than the variability of intensity signals, as demonstrated by analysing line profiles across the sample (Fig. 3g,h). By superimposing the data extracted from all the intensity and PLIM images, we observed no correlation between intensity signals and lifetime (Fig. S2). The higher uniformity of lifetime values and their independence from the recorded phosphorescence signals indicate that lifetime was not affected by the variability in tissue staining efficiency, as expected^33^. Bearing this in mind, we conducted PLIM of brain cortex slices (~ 1 cm^2^, ~ 1 mm thick), freshly dissected and surface-stained with NanO2-IR (Fig. 3i). The observed lifetime values (ranging ~ 20–25 µs) suggested that differences in local oxygenation are driven mainly by respiratory activity of particular areas of the tissue.

Based on these results, we concluded that surface staining of the two tissue types with NanO2-IR works reasonably well, and the PLIM imager enables fast visualisation of O_2_ levels at tissue/media interface. As expected, they were close to ambient O_2_ levels, with the interquartile range ~ 125–175 µM in the brain and ~ 170–200 µM in the lung (Fig. S1a). The magnitude of responses to mitochondrial modulators was proportional to respiratory activity of the tissue.

### Deep tissue staining and imaging using NanO2-IR probe

**Figure 4:**
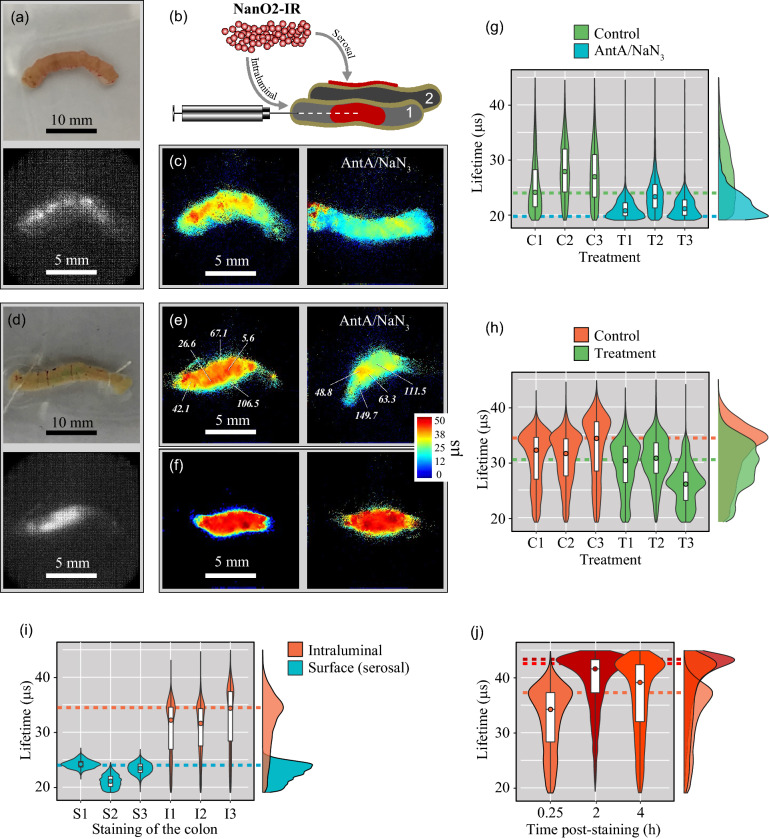
PLIM of O_2_ in the intraluminally stained mouse intestine samples. Photographs and phosphorescence intensity images of the small (**a**) and large (**d**) intestine stained for 15 min with NanO2-IR by intraluminal injection (1 mg/ml, 1 µl) as shown in (**b**); a separate set of samples was surface-stained in parallel. Typical PLIM images of the small (**c**) and large (**e**) intestine in the respiring state (left) and upon mitochondrial inhibition for 15 min by 10 µM AntA and 0.1% NaN_3_ (right). In (**e**), selected localised O_2_ concentrations (in μM) are shown in italic (see also Figs. S1 and S3). (**f**) PLIM images of the colon sample (**e**), collected 2 h and 4 h after the NanO2-IR injection. Lifetime frequency histograms in samples of the small (**g**) and large intestine (**h**) with normal and inhibited mitochondrial respiration. (**i**) Comparative analysis of lifetime frequencies in the colon samples stained with NanO2-IR intraluminally and topically on the serosal side. (**j**) Lifetime frequency histograms corresponding to the colon sample are shown in (**e**, **f**): lifetime images are taken 15 min, 2 h and 4 h post injection. PLIM images are prepared using rainbow false colours. In (**g**–**j**), violin plots show the distribution of lifetime values along the Y-axis against relative frequencies, with which these values were recorded in individual samples (X-axis). Box-plot partition of the violin plots demonstrates the median (circle) and interquartile ranges. Dashed horizontal lines show the aggregated modes per tissue type / staining protocol / treatment. On the right side, semi-transparent colour-matched aggregated lifetime distributions are shown. Colours correspond to the predominant spectrum in the PLIM images.

To probe O_2_ levels deeper inside tissue, we injected 1 µl of NanO2-IR (1 µg of the probe) into the lumen of excised samples of large and small mouse intestine (Fig. 4). In this case, the probe was shielded from the detector by a 0.2–0.25 mm thick intestinal wall^54^. It was contained inside the intestine and reported on O_2_ levels in the lumen and at the surface of mucosa. A separate series of intestinal samples were also surface-stained and imaged as described above.

Comparison of the PLIM images recorded from the mucosal and serosal sides of the small and large intestine revealed pronounced differences. For the intraluminally stained samples, lifetime distribution histogram was markedly shifted towards larger values compared to the surface-stained tissue (for the colon Δ-mode = 10.45 µs, Fig. 4i, Table S2). This indicated, as expected, a lower oxygenation of the inner areas of the intestine (Fig. S1b). The intraluminally stained samples with inhibited mitochondrial respiration showed largely reduced lifetime values, compared to the untreated samples (Δ-mode = 4.45 µs and 3.93 µs for the small and large intestine, respectively, Fig. 4c,e, Table S2). Still, lifetime signals were not uniform and did not correspond to fully oxygenated state (see calibration in Fig. 2), most likely because respiration in the surrounding tissue was not fully inhibited by the slowly diffusing drugs. Non-mitochondrial, AntA/NaN_3_-independent respiration could also contribute to the residual tissue deoxygenation^55^. For the colonic tissue samples shown in Fig. 4e, detailed oxygenation maps were produced (Fig. S3).

Comparing the non-treated intraluminally stained samples of the two anatomically different areas of the intestine, we found larger lifetime values for the colon compared to the small intestine, suggesting deeper colonic deoxygenation (Δ-mode = 10.13 µs, Fig. 4c,e,g,h).

Aiming to examine the stability of lifetime signals and respiratory activity of the intestinal tissue, we also conducted time-lapse PLIM analysis of intraluminally stained colon samples over 4 h. We observed a marked increase in lifetime values between 15 min and 2 h incubation (Δ-mode = 6.09 µs), which reflected the reduction in O_2_ levels in the luminal part of the intestine. No further changes in lifetime values / oxygenation occurred between 2 and 4 h of incubation. Indeed, the mode value of ~ 43 µs corresponded to almost complete deoxygenation (~ 1 µM O_2_), with Δ-O_2_ =  ~ 140 µM between the serosal and mucosal sides. We think that the lower lifetime values at 15 min reflect partial reoxygenation of the lumen during NanO2-IR injection with a Hamilton needle (Ø = 0.72 mm), which transiently enabled O_2_ access to the lumen (Fig. 4b,e,f,j). From the fact that O_2_ levels remained very low for at least 4 h, we also concluded that tissue sustained respiration over the whole period of manipulation, including the dissection, preparation, staining, incubation and imaging procedures.

**Figure 5:**
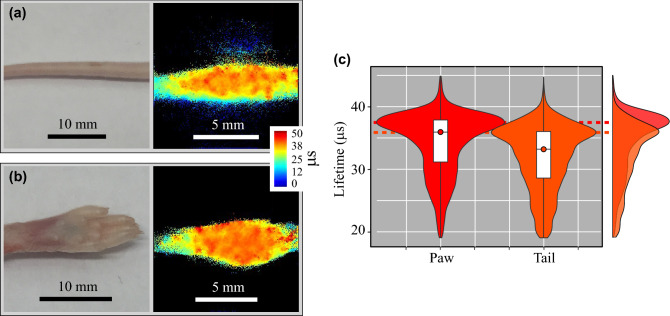
PLIM of O_2_ in the subcutaneously stained tail (**a**) and paw (**b**) of a mouse (post mortem). On the left, photographs and PLIM images are shown. Rainbow false colours demonstrate the range of lifetime values. (**c**) Lifetime frequency distribution calculated from (**a**) and (**b**); violin plots show the distribution of lifetime values along the Y-axis, and relative frequencies, with which these values were obtained (X-axis). Box-plot partition of the violin plots demonstrates the median (circle) and interquartile ranges. Dashed horizontal lines show the aggregated modes for each tissue type. On the right side, semi-transparent colour-matched aggregated lifetime distributions are shown.

We also applied subcutaneous injection of the NanO2-IR probe into intact tissue of freshly sacrificed mice. The selected parts of the tail and paw were thicker (~ 0.5 mm^56,57^) and less permeable to light than intestinal wall. As a result, our first attempt with 1 µl injection of NanO2-IR did not produce sufficient signals under the same imaging settings as above. However, when injection volume was increased to 5 µl (5 µg of the probe), NanO2-IR was successfully imaged and PLIM produced high lifetime values both in the tail and the paw (Fig. 5, Table S2). These PLIM results are in agreement with the current knowledge about deep or even complete deoxygenation of tissues with interrupted O_2_ supply, as exemplified by the brain tissue after clinical death^58^.

## Conclusions

The Tpx3Cam based PLIM macro-imager was demonstrated in fast, high-resolution imaging of O_2_ in sizeable biological samples, including liquid samples with respiring cells, live post-mortem animal tissue and whole organs, passively stained with NanO2-IR probe on the surface or microinjected in tissue depth. Tissue staining and PLIM protocols were optimised to ensure no detectable adverse effects of NanO2-IR probe on the live tissue samples during imaging (within 1 h), and tissues respiration remained active for at least 4 h after isolation. The imager allowed accurately measuring probe lifetime values and generating detailed O_2_ concentration maps for tissue surface/interface and inner areas, the latter imaged through 0.2–0.5 mm thick tissue layers under low-power LED excitation. Along with single photon sensitivity, advantages of our platform also include flexibility, transportability, high level of technology integration and relatively low cost (< 30% of the price for standard PLIM systems). The imager was able to reveal an adequate span of measurable O_2_ levels (0–210 µM) in ex vivo tissue samples with higher (e.g. the lungs or brain surface) and lower (e.g. colonic lumen or inner areas of the paw and tail) O_2_ levels than those typically found in vivo in vascularised tissues. Potential usability of the imager in various physiological in vivo studies is also supported by the fact that it can accurately measure expected changes in probe lifetime signal: decreases upon mitochondrial inhibition or increases after moving deeper into tissue.

## Supplementary information

Supplementary Information
