# Supplementary material for: Mapping O2 concentration in ex-vivo tissue samples on a fast PLIM macro-imager
Source: Sci Rep. 2020 Nov 4;10:19006. doi: 10.1038/s41598-020-75928-3 (PMC7642408; doi:10.1038/s41598-020-75928-3)
Supplement: Supplementary file 1 — Supplementary Information [file 41598_2020_75928_MOESM1_ESM.pdf]

Mapping O_2 concentration in *ex-vivo* tissue samples on a fast PLIM macro-imager

Rajannya Sen[1#], Alexander V. Zhdanov[1#], Thomaz F.S. Bastiaanssen[2,3], Liisa M. Hirvonen[4], Peter Svihra[5,6], Patrick Fitzgerald[2], John F. Cryan[2,3], Stefan Andersson-Engels[7], Andrei Nomerotski[8], Dmitri B. Papkovsky[1*]

[1]School of Biochemistry and Cell Biology, University College Cork, Cork, Ireland

[2]APC Microbiome Ireland, University College Cork, Ireland

[3]Department of Anatomy and Neuroscience, University College Cork, Ireland

[4]Centre for Microscopy, Characterisation and Analysis (CMCA), the University of Western Australia, Crawley WA 6009, Australia

[5]Department of Physics, Faculty of Nuclear Sciences and Physical Engineering, Czech Technical University, Prague 115 19, Czech Republic

[6]Department of Physics and Astronomy, School of Natural Sciences, the University of Manchester, Manchester M139PL, United Kingdom

[7]Irish Photonics Integration Centre, Tyndall National Institute, Cork, Ireland

[8]Physics Department, Brookhaven National Laboratory, Upton, New York, 11973 USA

SUPPLEMENTARY INFORMATION

Supplementary Table S1. Exemplary/typical PLIM acquisition parameters used for the different tissue types and staining methods.

Tissue (staining method)	Treatment	Image collection time (sec)	Total frame number	Triggered pixels number	Readout rate (MHz)	Photon events number	Event size range	Undersize events number	Oversize events number	Photon count rate (MHz)
Cortex (outer surface)	No	20	99210	52803332	2.6	9364458	3-15	704703	1066970	0.5
	AntA / NaN_3	20	99923	38134789	1.9	7196821	3-15	489437	628022	0.4
Lungs (outer surface)	No	20	99453	22779858	1.1	4425511	3-15	355928	336448	0.2
	AntA / NaN_3	20	100014	25072928	1.3	4938119	3-15	404726	361494	0.2
Large intestine (intraluminal)	No	20	100120	28378586	1.4	4713630	3-15	365106	572190	0.2
	AntA / NaN_3	20	100185	24924614	1.2	4226943	3-15	355015	479318	0.2
Small intestine (intraluminal)	No	20	100028	23500000	1.2	4052414	3-15	347450	433438	0.2
	AntA / NaN_3	20	100141	37126634	1.9	6044806	3-15	476891	797582	0.3

Supplementary Table S2. Characteristics of lifetime values measured ex vivo in mouse tissues

Tissue / Staining	Sample /Treatment	Median	Interquartile range	Mode
Brain / surface-stained	C1	24.25	3.94	23.42
	C2	23.78	2.4	
	C3	23.08	3.36	
	T1 / AntA/NaN_3	NA	NA	19.93
	T2 / AntA/NaN_3	23.08	1.54	
	T3 / AntA/NaN_3	21.02	2.07	
Lung / surface stained	C1	21.32	2.08	20.51
	C2	20.27	1.53	
	C3	19.67	0.9	
	T1 / AntA/NaN_3	NA	NA	19.05
	T2 / AntA/NaN_3	19.05	0.05	
	T3 / AntA/NaN_3	19.42	0.3	
Small intestine / intraluminal	C1	24.2	6.44	24.26
	C2	27.96	7.54	
	C3	26.92	7.58	
	T1 / AntA/NaN_3	21.06	2.39	19.81
	T2 / AntA/NaN_3	23.56	4.27	
	T3 / AntA/NaN_3	20.91	2.96	
Large intestine / intraluminal	C1	32.13	7.65	34.39
	C2	31.53	6.77	
	C3	34.28	8.99	
	T1 / AntA/NaN_3	30.21	6.62	30.46
	T2 / AntA/NaN_3	30.68	5.55	
	T3 / AntA/NaN_3	25.95	5.18	
Large intestine / surface-stained *vs* intraluminal	S1	24.13	1.01	23.95
	S2	21.02	1.83	
	S3	23.4	1.5	
	I1	32.13	7.65	34.39
	I2	31.53	6.77	
	I3	34.28	8.99	
Large intestine time lapse	15 min	34.28	8.99	37.2
	2 h	41.68	6.05	43.29
	2 h	39.2	10.39	42.5
Paw-Tail	Paw	35.99	6.76	37.57
	Tail	33.23	7.47	35.97

$W = 1598807280$, $p < 2.2E^{-16}$

$W = 635409826$, $p < 2.2E^{-16}$

$W = 429612$, $p < 2.2E^{-16}$

$W = 426940586$, $p < 2.2E^{-16}$

$W = 166178566$, $p < 2.2E^{-16}$

$W = 270684296$, $p < 2.2E^{-16}$

$W = 78422474$, $p < 2.2E^{-16}$

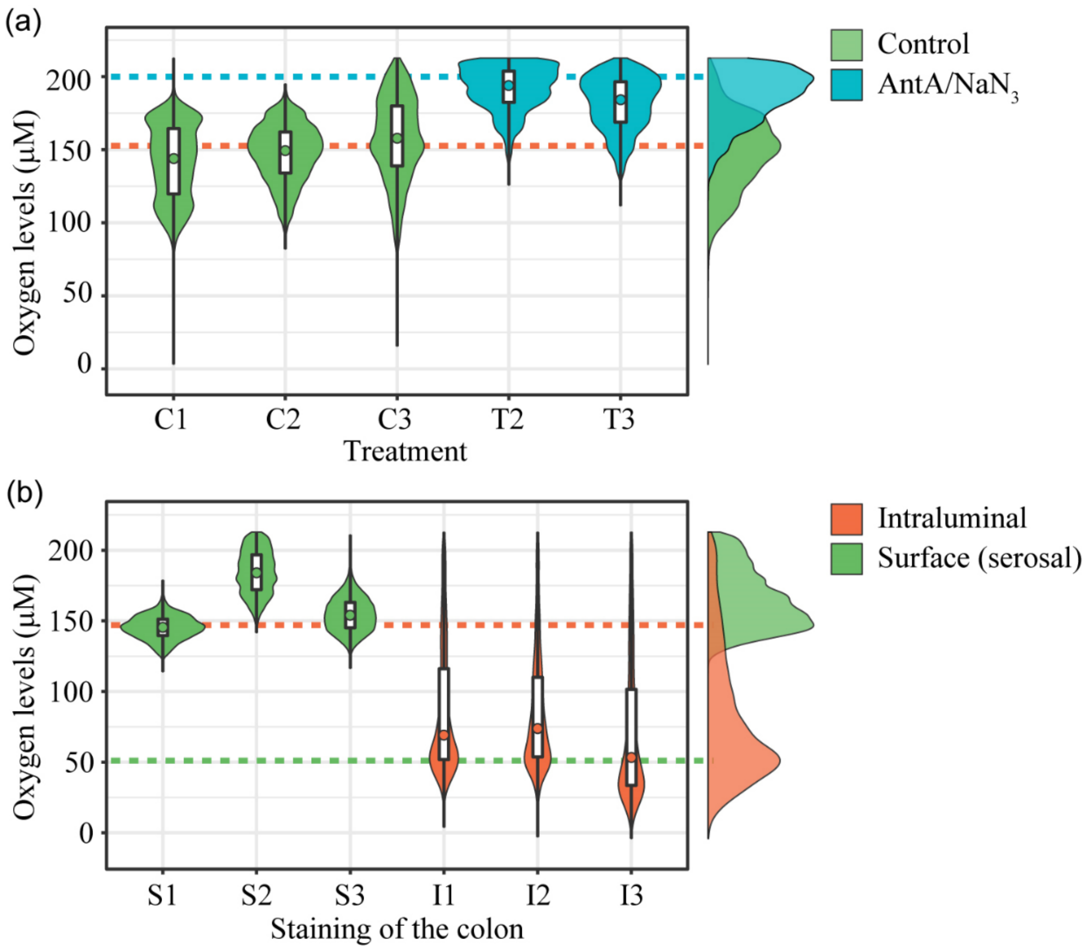

Supplementary Figure S1. Imaging of oxygenation in ex-vivo samples of mouse tissue: effects of tissue type, treatment and O_2 availability. (a) O_2 levels in the normally respiring brain tissue surface-stained with NanO2-IR, and in the tissue with inhibited mitochondrial respiration (AntA/NaN_3). (b) O_2 levels on the outer, serosal side of the colon (surface-stained) and inside the lumen or on the inner, mucosal surface. O_2 levels are calculated from lifetime values shown it Fig. 3e and Fig. 4i. Violin plots show: on the Y-axis - the distribution of O_2 values; on the X-axis - relative frequencies of given O_2 values. Colours correspond to the predominant spectrum in the PLIM images. Box-plot partition of the violin plots represents the median and interquartile ranges. Dashed horizontal lines represent the aggregated modes for the different tissue type, treatment or staining method. On the right side, semi-transparent colour-matched aggregated distributions are shown.

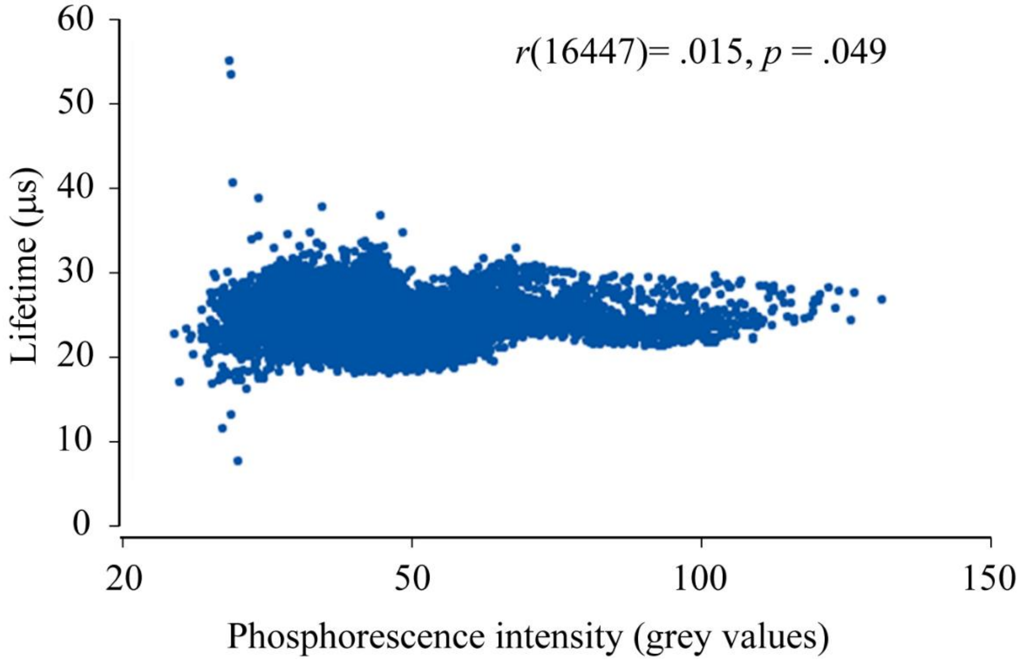

Supplementary Figure S2. Dependence of the calculated lifetime values on measured phosphorescence intensity signals. Pearson correlation analysis of the PLIM and intensity images of a surface-stained brain sample.

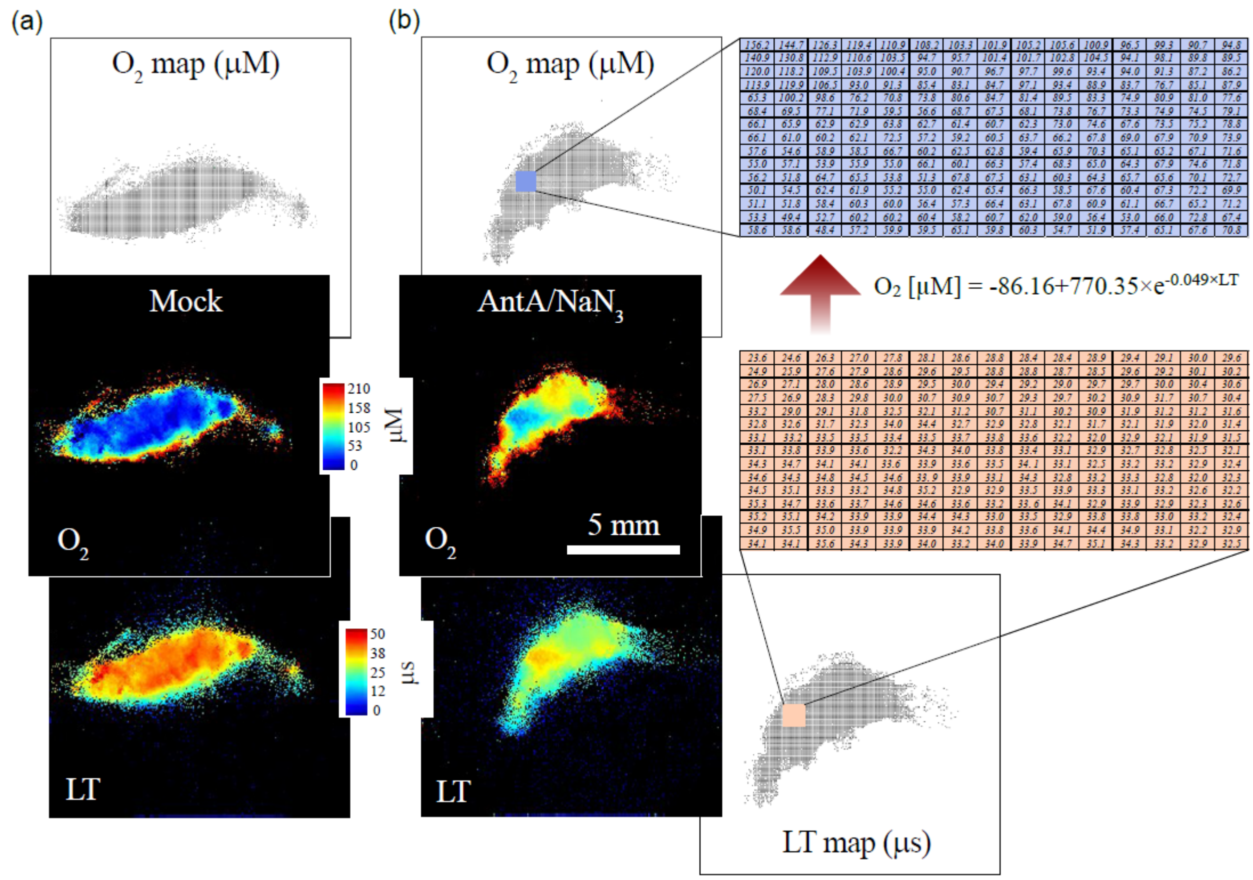

Supplementary Figure S3. Mapping O_2 in the lumen of mouse colon ex vivo. (a) From bottom to top, the lifetime (LT) image, O_2 image and detailed O_2 map of the lumen of mouse colon stained as described for Fig. 4e. (b). Similar to (a) data set obtained using mouse colon treated for 15 min with AntA and NaN_3. Right panel in (b) also shows the LT map and magnified fragments of LT and O_2 maps that show the conversion of LT values (μs) into O_2 concentrations (μM) using the indicated analytical equation.
